# Using allometric procedures to substantiate the plastochrone method for eelgrass leaf growth assessments

**DOI:** 10.1186/1742-4682-10-34

**Published:** 2013-05-16

**Authors:** Héctor Echavarría-Heras, Elena Solana-Arellano, Cecilia Leal-Ramírez, Oscar Castillo

**Affiliations:** 1 Centro de Investigación Científica y de Educación Superior de Ensenada, carretera Ensenada-Tijuana No. 3918, Zona Playitas, Código Postal 22860. Apdo. Postal 360, Ensenada, BC, Mexico; 2 Tijuana Institute of Technology, Tijuana, Baja California, Mexico

**Keywords:** Eelgrass, Allometric scaling, Leaf growth, Plastochrone method, Formal validation

## Abstract

Estimation of leaf productivity in eelgrass (*Zostera marina L*.) is crucial for evaluating the ecological role of this important seagrass species. Although leaf marking techniques are widely used to obtain estimates of leaf productivity, the accuracy of these assessments, has been questioned mainly because these fail to account for leaf growth bellow the reference mark and also because they apparently disregard the contribution of mature leaf tissues to the growth rate of leaves. On the other hand, the plastochrone method is a simpler technique that has been considered to effectively capture growth in a more realistic way, thereby providing more accurate assessments of both above- and below-ground productivities. But since the actual values of eelgrass growth rates are difficult to obtain, the worth of the plastochrone method has been largely vindicated because it produces assessments that overestimate productivity as compared to estimates obtained by leaf marking. Additionally, whenever eelgrass leaf biomass can be allometrically scaled in terms of matching leaf length in a consistent way, the associated leaf growth rates can be also projected allometrically. In this contribution, we used that approach to derive an authentication of the plastochrone method and formally demonstrate that, as has been claimed to occur for leaf marking approaches, the plastochrone method itself underestimates actual values of eelgrass leaf growth rates. We also show that this unavoidable bias is mainly due to the inadequacy of single-leaf biomass assessments in providing a proxy for the growth of all leaf tissue in a shoot over a given interval. Moreover, the derived formulae give conditions under which assessments of leaf growth rates using the plastochrone method would systematically underestimate matching values obtained by leaf marking procedures. And, assessments of leaf growth rates obtained by using the present data show that plastochrone method estimations underestimated corresponding proxies obtained allometrically (27%), or through leaf marking (35%). Allometric projection is recommended as a simpler and more effective procedure to reduce the bias in eelgrass leaf productivity estimations that associates to the use of plastochrone methods.

## Introduction

Eelgrass (*Zostera marina*) is a cosmopolitan seagrass species that plays an important role in shallow and nearshore ecosystems. This temperate macrophyte is distributed in Northern Hemisphere habitats from the Arctic Circle to the Tropic of Cancer [1], where it provides a nursery for fish and as a substrate for attached algae and epifauna [2,3]. By fixing large amounts of carbon through photosynthesis, eelgrass plays an important trophic role, sustaining detrital food chains and other secondary producers [4]. Eelgrass also helps in the remediation of contaminated sediments [5] by filtering and retaining nutrients from the water column [6] and contributing to the stabilization of sediments [7]. Moreover, eelgrass meadows reduce erosional forces by stumping wave energy, thus promoting the stabilization of adjacent shorelines [8,9].

The variability in eelgrass biomass constitutes a dynamic link between its structural and trophic roles, because changes in the amount of organic carbon that can be fixed modulate the structure of the habitat for the associated biota. These organisms are affected in different ways when changes in biomass occur seasonally or unpredictably [10]. Therefore, accurate measurements of the standing crop and productivity of eelgrass constitute an important input for evaluating the ecological functions and values of this significant seagrass species [11].

Several methods have been developed to measure seagrass growth and productivity [12-14]. Procedures for measuring productivity in aquatic macrophytes have included the assessment of changes in biomass over a growing season, as well as the quantification of oxygen production or incorporation of ^14^C during photosynthesis. But the accuracy of these methodologies was questioned early on [15-18]. Growth in seagrasses occurs through the expansion of modules formed by rhizome segments, which have bundles of attached leaves and roots. Because every leaf produced corresponds to the production of a rhizome node, it is reasonable to assume that seagrass growth and leaf formation are equivalent processes [19]. This conspicuous feature has encouraged efforts to estimate the growth of eelgrass, as well as that of other seagrasses with ribbon-like leaves, by measuring leaf growth. Leaf growth in seagrasses can be estimated by using the leaf marking technique which was originally proposed as a way of avoiding the underestimation of growth when using oxygen metabolism measurements [16,18]. Leaves were marked above the sheath with a small staple at the height of a reference frame placed above the sediment, and then recovered after a period of 2–4 weeks. The new growth in each leaf between the reference frame and the staple was then weighed. The ratio of this weight to the number of days of growth determined the leaf growth rate. Because this procedure is considered to give consistent estimations [19,20] it has been used and modified by several authors (e.g. [21-23]).

For assessment of growth in *Zoostera marina* in particular, workers have marked blades using felt-tip pens, staples and hypodermic needles [4,24-26], with the reference level being either the leaf tip [25,27] or a point at the top of the sheath [12,24,26,28]. However, Brouns [29] asserted that this technique only estimates blade production and is not suitable for measuring total production. The reliability and accuracy of the leaf-marking method has been also questioned by others; for example, because it does not account for new leaf growth within the sheath below the reference mark [24], or it might inflict damage to tissues [19], or fail to account for leaf maturation processes [11,13,30], or that it requires destructive dry-weight measurements.

Based on the observation that the production of every seagrass leaf is associated with the production of a rhizome node, Patriquin [31] envisioned that seagrass root and rhizome production can be also estimated by determining the time interval for the formation of new leaves and by counting the associated leaf scars. The time elapsed between the appearance of two consecutive leaves has been termed a plastochrone interval or plastochrone index [32,33]. For eelgrass, Jacobs [4] used the average weight of the third leaf on sampled shoots as a substitute for the leaf biomass gained by a shoot over a plastochrone interval and divided this leaf biomass surrogate by the value of the plastochrone interval to assess growth rates of leaves. Gaeckle and Short [11] endorsed the method of Jacobs [4] and used the weight of a mature leaf to represent all growing leaf tissue in a shoot over a given a plastochrone interval to calculate eelgrass growth, calling the leaf-biomass to plastochrone ratio the plastochrone method for eelgrass leaf-growth assessments. And Gaeckle and Short [11] concluded that leaf marking results in lower estimates of leaf growth than the plastochrone-based method, as asserted by Brouns [29], and they considered that this approach both fully captures growth and is also simpler and non-destructive.

But despite the advantages of plastochrone methods, their use has not been yet formally substantiated. In the present research we attempt to fill this gap by using allometric models and a discrete mathematical formulation for the increment in biomass gained by an eelgrass leaf over a time interval to derive an equation which expresses the mean shoot leaf-growth rates in terms of a factor of the leaf biomass to plastochrone ratio plus a remainder, and explore their analytical implications. And, we extend aforementioned result to provide a formal device aimed at obtaining the absolute deviations between leaf growth-rate assessments estimated by using the conventional leaf-marking technique and those obtained by using the plasthochrone method. We also corroborate the derived formulae using both simulation and real data. Finally, we stress the advantages of using allometric methods in eelgrass research and discuss the findings of this study.

## Data and related calculations

The data used for this study were collected in a *Z. marina* meadow in Punta Banda Estuary, located near Ensenada, Baja California, Mexico. Form March 1999 to July 2000 we visited the site biweekly. At each sampling time using the Kentula and McIntire technique [26] approximately 40 shoots were marked, and those previously marked were retrieved. On all the shoots collected, the sheaths were peeled off and the leaf contents were separated. We measured the lengths of each of the retrieved leaves and the associated leaf-length increments gained over the marking interval. The matching biomasses were obtained by direct dry weight determinations. The associated *in situ* values for the mean-shoot leaf growth over the marking interval were estimated directly by dividing total leaf production in the marked shoots retrieved by the number of days elapsed.

Matching estimations, produced by the leaf marking method, were obtained by subtracting leaf production within the sheath to the *in situ* rates. Total leaf productivity in retrieved shoots was also determined indirectly by the allometric approach described in equation (12a) below, and also by applying the plastochrone method.

## A formal derivation of the plastochrone method

In this section we show how, using allometric models for the representation of eelgrass leaf biomass in terms of length, we can provide a formal substantiation of the plasthocrone method for leaf-growth assessments. With that aim, we use the index *s* to label a generic *Zostera marina* shoot and let n(*s*) stand for the number of leaves it holds. This number includes wholly formed leaves attached to the sheath and those developing inside this structure. We let *l*_*js*_(*t*) stand for the length of *j-th* leaf in the addressed shoot, and use *∆t* to denote a positive amount of time. Thus, the symbol *∆l*_*js*_(*t, ∆t*) stands for the increment in length attained by the leaf *l*_*js*_(*t*) along the interval [*t, t + ∆t*], i.e.

(1) $$∆l_{\mathrm{js}}\left(t,∆t\right)=l_{\mathrm{js}}\left(t+∆t\right)-l_{\mathrm{js}}\left(t\right)$$

Correspondingly, the symbol *ω*_*js*_(*t*) with  1 ≤ *j* ≤ *n*(*s*) denotes the dry weight at a time *t* of the *j*-*th* leaf in the considered shoot. Also, we will let *∆ω*_*js*_(*t*, *∆t*)  denote the gain in dry weight for *l*_*js*_(*t*) occurring along the interval [*t, t + ∆t*], i.e.,

(2) $$∆\omega_{\mathrm{js}}\left(t,∆t\right)=\omega_{\mathrm{js}}\left(t+∆t\right)-\omega_{\mathrm{js}}\left(t\right)$$

Let’s denote by means of the symbol *Lg*_*s*_(*t*, ∆*t*) the mean leaf growth rate attained by a shoot *s* during the interval [*t,t *+ ∆*t*]. We then have the mean shoot leaf-growth rates

(3) $$Lg_{s}\left(t,∆t\right)=\underset{j}{\sum }\frac{∆\omega_{\mathrm{js}}\left(t,∆t\right)}{∆t}$$

Similarly the symbol *Lg*(*t*, *∆t*) represents the average of the *Lg*_*s*_(*t*, *∆t*) values taken over the number of shoots collected at a time *t* + *∆t*, i.e.,

(4) $$\mathrm{Lg}\left(t,∆t\right)=\frac{\sum_{s}Lg_{s}\left(t,∆t\right)}{N\left(t+∆t\right)}$$

Where *N*(*t* + ∆*t*) is the number of shoots collected at a time *t* + ∆*t*. In what follows the calculated values for the *Lg*(*t*, ∆*t*) rates will be referred to as *in situ* mean leaf-growth rates.

Assuming that there are parameters *α* and *β* such that *ω*_*j*s_(*t*) and *l*_*js*_(*t*) are linked through the allometric equation

(5) $$\omega_{\mathrm{js}}\left(t\right)=\alpha l_{\mathrm{js}}{\left(t\right)}^{\beta }$$

then a related expression for the leaf biomass increment *∆ω*_*js*_(*t*, *∆t*) can be derived [34] and this becomes

(6) $$∆\omega_{\mathrm{ajs}}\left(t,∆t\right)=\alpha l_{\mathrm{js}}{\left(t+∆t\right)}^{\beta }\;\delta_{\mathrm{js}}\left(t,∆t\right)$$

with

(7) $$\delta_{\mathrm{js}}\left(t,∆t\right)=\left(1-{\left(1-\frac{∆l_{\mathrm{js}}\left(t,∆t\right)}{l_{\mathrm{js}}\left(t+∆t\right)}\right)}^{\beta }\right)$$

And, since we have

(8) $$0\leq ∆l_{\mathrm{js}}\left(t,∆t\right)\leq l_{\mathrm{js}}\left(t+∆t\right)$$

we must also have,

(9) $$0\leq \delta_{\mathrm{js}}\left(t,∆t\right)\leq 1$$

Since the result of equation (6) is derived from equation (5) we must formally have

(10) $$∆\omega_{\mathrm{js}}\left(t,∆t\right)=∆\omega_{\mathrm{ajs}}\left(t,∆t\right)$$

Nevertheless, for applications to a given data set, when calculating, the leaf biomass increments of equation (6) we must rely on estimates for the parameters *α* and *β*, hence the associated uncertainties will set,

(10a) $$∆\omega_{\mathrm{js}}\left(t,∆t\right)=∆\omega_{\mathrm{ajs}}\left(t,∆t\right)+R_{\mathrm{js}}^{\mathrm{wa}}\left(t,∆t\right)$$

where $\;R_{\mathrm{js}}^{\mathrm{wa}}\left(t,∆t\right)$, is a residual term.

Correspondingly we will denote by means of the symbol *Lga*_*s*_(*t*, *∆t*) the allometric surrogate of the *Lg*_*s*_(*t*, ∆*t*) rates of equation (3). That is,

(11) $$\mathrm{Lg}a_{s}\left(t,∆t\right)\;=\underset{j}{\sum }\frac{∆\omega_{\mathrm{ajs}}\left(t,∆t\right)\;}{∆t}$$

Again we must formally have,

(11a) $$Lg_{s}\left(t,∆t\right)=\mathrm{Lg}a_{s}\left(t,∆t\right)$$

and, as it was elaborated in equation (10) when estimates of the allometric parameters are used to calculate the *∆ω*_*ajs*_(*t*, *∆t*) proxies we must have,

(11b) $$Lg_{s}\left(t,∆t\right)=\mathrm{Lg}a_{s}\left(t,∆t\right)+R_{s}^{\mathrm{wa}}\left(t,∆t\right),$$

where $R_{s}^{\mathrm{wa}}\left(t,\mathrm{\Delta t}\right)$ is the involved estimation error.

Averaging the *Lga*(*t*, ∆*t*) values over the number of shoots collected at time *t* + ∆*t*, one gets allometrically projected values *Lag*(*t*, ∆*t*) for the *in situ* leaf growth rates *Lg*(*t*, ∆*t*) of equation (4), these are

(12a) $$\mathrm{Lga}\left(t,\mathrm{\Delta t}\right)=\frac{\sum_{s}\mathrm{Lg}a_{s}\left(t,\mathrm{\Delta t}\right)}{\;N\left(t+\mathrm{\Delta t}\right)}.$$

Furthermore we must formally have,

(12b) $$\mathrm{Lg}\left(t,\mathrm{\Delta t}\right)=\mathrm{Lga}\left(t,\mathrm{\Delta t}\right)+\mathrm{Ba}\left(t,\mathrm{\Delta t}\right)$$

where the term *Ba*(*t*, ∆*t*) stand for the pertinent approximation bias. Again, only in the case in which both equations (10) and (11a) hold we will have that the *Lg*(*t*, ∆*t*) rates of equation (4) and their allometric representations *Lga*(*t*, ∆*t*) are equivalent, this setting *Ba*(*t*, ∆*t*) = 0. In what follows the result of equation (12a) will be called “allometric method for the projection of eelgrass leaf growth rates” or simply as “allometric method”.

Now let *p*(*t*, ∆*t*) denote the value of the leaf plastochrone interval calculated using the leaf marking data resultant for the interval [*t*, *t* + ∆*t*] [4], and let’s assume that the biomass of the third leaf on each retrieved shoot provides a substitute for all growing leaf tissue in that shoot along a time interval of size *p*(*t*, ∆*t*) [4,11]. Then, in accordance with our convention, the biomass of this leaf will be denoted by means of  *ω*_3*s*_(*t*, *Δt*), and let’s also represent the resulting leaf biomass to plastochrone ratio, using the symbol *Lgp*_*s*_(*t*, ∆*t*) that is,

(13) $$\mathrm{Lg}p_{s}\left(t,\mathrm{\Delta t}\right)=\frac{\omega_{3s}\left(t,\mathrm{\Delta t}\right)}{p\left(t,\mathrm{\Delta t}\right)}$$

this equation is the formal representation of the plastochrone method, which is considered to yield the mean rate of growth for all the leaves on a retrieved shoot *s* and over the interval [*t*, *t* + ∆*t*] [4,11,14]. Then, averaging the shoot rate values *Lgp*_*s*_(*t*, ∆*t*) over the number of shoots collected at a time *t* + ∆*t* one obtains the proxy values *Lgp*(*t*, ∆*t*) produced by the plastochrone method and corresponding to the *in situ* leaf growth rate values *Lg*(*t*, ∆*t*), they are,

(14a) $$\mathrm{Lgp}\left(t,\mathrm{\Delta t}\right)=\frac{\sum_{s}\mathrm{Lg}p_{s}\left(t,\mathrm{\Delta t}\right)}{N\left(t+\mathrm{\Delta t}\right)}.$$

Again by letting *Bp*(*t*, ∆*t*) stand for the associated bias we have,

(14b) $$\mathrm{Lg}\left(t,\mathrm{\Delta t}\right)=\mathrm{Lgp}\left(t,\mathrm{\Delta t}\right)+\mathrm{Bp}\left(t,\mathrm{\Delta t}\right)$$

Now, assuming that equation (10) holds then from equations (11) and (13) one obtains

(15) $$Lg_{s}\left(t,\mathrm{\Delta t}\right)\;=\;C_{s}^{\mathrm{ap}}\;\left(t,\mathrm{\Delta t}\right)\mathrm{Lg}p_{s}\left(t,\mathrm{\Delta t}\right)+R_{s}^{\mathrm{ap}}\left(t,\mathrm{\Delta t}\right),$$

where the factor $C_{s}^{\mathrm{ap}}\;\left(t,\mathrm{\Delta t}\right)$ is given by,

(16) $$C_{s}^{\mathrm{ap}}\;\left(t,\mathrm{\Delta t}\right)=\frac{\delta_{3s}\left(t,\mathrm{\Delta t}\right)p\left(t,\mathrm{\Delta t}\right)}{\mathrm{\Delta t}},$$

and $R_{s}^{\mathrm{ap}}\left(t,\mathrm{\Delta t}\right)$ is a positive remainder defined through,

(17) $$R_{s}^{\mathrm{ap}}\left(t,\mathrm{\Delta t}\right)=\underset{j}{\sum }\frac{\delta_{\mathrm{js}}\left(t,\mathrm{\Delta t}\right)\omega_{\mathrm{js}}\left(t+\mathrm{\Delta t}\right)I\left(j,3\right)}{\mathrm{\Delta t}}$$

with,

(18) $$I\left(j,3\right)=\left\{\begin{array}{ccc} 0 & \mathrm{if} & j=3\\ 1 & & \mathrm{otherwise} \end{array}\right)\;$$

Since ω_*js*_(*t*, ∆*t*) is positive and we have also observed that *δ*_*js*_(*t*, ∆*t*) does not simultaneously vanish for all values of the index *j* in a retrieved shoot, we must have,

(19) $$R_{s}^{\mathrm{ap}}\left(t,\mathrm{\Delta t}\right)>0.$$

Also, from inequality (9) and equation (16) we obtain,

(20) $$\;0\leq C_{s}^{\mathrm{ap}}\;\left(t,\mathrm{\Delta t}\right)\leq \frac{p\left(t,\mathrm{\Delta t}\right)}{\mathrm{\Delta t}}$$

Then, since depending on the order relationship that *p*(*t*, ∆*t*) and ∆*t* satisfy, the ratio of *p*(*t*, ∆*t*) to ∆*t* can take any positive value, and since as given by equation (16) $\;C_{s}^{\mathrm{ap}}\;\left(t,\mathrm{\Delta t}\right)$ depends continuously on *δ*_*3s*_(*t*, ∆*t*), *p*(*t*, ∆*t*) and ∆*t* it can formally take any positive value. For instance we could set $C_{s}^{\mathrm{ap}}\;\left(t,\mathrm{\Delta t}\right)=1$, which according with equation (16) leads to the equivalent equation

(21) $$\delta_{3s}\left(t,\mathrm{\Delta t}\right)=\frac{\mathrm{\Delta t}}{p\left(t,\mathrm{\Delta t}\right)}$$

which taking into account inequality (9) could be only satisfied whenever the order relationship ∆*t* ≤ *p*(*t*, ∆*t*) holds.

Moreover, from equations (4), (13) and (15) one obtains,

(22) $$\mathrm{Lg}\left(t,\mathrm{\Delta t}\right)=\mathrm{Cap}\left(t,\mathrm{\Delta t}\right)\mathrm{Lgp}\left(t,\mathrm{\Delta t}\right)+\mathrm{Bap}\left(t,\mathrm{\Delta t}\right)$$

where

(23) $$\mathrm{Cap}\left(t,\mathrm{\Delta t}\right)=\frac{\sum_{s}C_{s}^{\mathrm{ap}}\;\left(t,\mathrm{\Delta t}\right)\mathrm{Lg}p_{s}\left(t,\mathrm{\Delta t}\right)}{\sum_{s}\mathrm{Lg}p_{s}\left(t,\mathrm{\Delta t}\right)\;}$$

and

(24) $$\mathrm{Bap}\left(t,\mathrm{\Delta t}\right)=\frac{\sum_{s}\;R_{s}^{\mathrm{ap}}\left(t,\mathrm{\Delta t}\right).}{N\left(t+\mathrm{\Delta t}\right)\;}$$

so, inequality (19) implies,

(25) $$\mathrm{Bap}\left(t,\mathrm{\Delta t}\right)>0,$$

and using inequality (20) and equation (23) we get,

(26) $$0\leq \mathrm{Cap}\left(t,\mathrm{\Delta t}\right)\leq \frac{p\left(t,\mathrm{\Delta t}\right)}{\mathrm{\Delta t}}.$$

Again, since $C_{s}^{\mathrm{ap}}\;\left(t,\mathrm{\Delta t}\right)$ varies continuously and *Lgp*_*s*_(*t*, ∆*t*) does not vanish and depends continuously on both ω_*3s*_(*t*, ∆*t*) and *p*(*t*, ∆*t*) from equation (23) we conclude that *Cap*(*t*, ∆*t*) varies continuously and takes all the positive values within the range set by inequality (26).

From equation (22) we can formally infer that the equivalence of *Lg*(*t*, ∆*t*) and *Lgp*(*t*, ∆*t*) would occur whenever the constrains *Bap*(*t*, ∆*t*) = 0 and *Cap*(*t*, ∆*t*) = 1 simultaneously hold. Moreover from equation (23) we have that *Cap*(*t*, ∆*t*) = 1 is equivalent to the equation,

(27) $$\underset{s}{\sum }\left(C_{s}^{\mathrm{ap}}\;\left(t,\mathrm{\Delta t}\right)-1\right)\mathrm{Lg}p_{s}\left(t,\mathrm{\Delta t}\right)=0$$

which will be satisfied whenever $C_{s}^{\mathrm{ap}}\;\left(t,\mathrm{\Delta t}\right)=1$ and which according to the elaboration sustaining equation (21) can only occur whenever the order relationship ∆*t* ≤ *p*(*t*, ∆*t*) holds. Indeed from inequality (20) we have,

(28) $$-1\leq C_{s}^{\mathrm{ap}}\;\left(t,\mathrm{\Delta t}\right)-1\leq \frac{p\left(t,\mathrm{\Delta t}\right)-\mathrm{\Delta t}}{\mathrm{\Delta t}},$$

then if we assumed that *Δt* > *p*(*t*, *Δt*)  the factor ($\;C_{s}^{\mathrm{ap}}\;\left(t,\mathrm{\Delta t}\right)-1)$ in equation (27) would not change signs, and since *Lgp*_*s*_(*t*, ∆*t*) is positive for all values of *t* equation (27) could not be satisfied. But even though, as we have elaborated above, whenever ∆*t* ≤ *p*(*t*, ∆*t*), the statement *Cap*(*t*, *Δt*) = 1 *Cap*(*t*, ∆*t*) = 0 could be formally satisfied, inequality (25) implies that the equation *Bap*(*t*, ∆*t*) = 0 could not be satisfied, precluding the equivalence of *Lg*(*t*, ∆*t*) and *Lgp*(*t*, ∆*t*). As a result, when projecting the *Lg*(*t*, ∆*t*) rates by means of the *Lgp*(*t*, ∆*t*) proxy values, we can anticipate a systematically positive or negative bias. This direct bias has been denoted by means of *Bp*(*t*, ∆*t*), (cf. eq. 14b) and from equation (22) it is given by

(29) $$\mathrm{Bp}\left(t,\mathrm{\Delta t}\right)\;=\left(\mathrm{Cap}\left(t,\mathrm{\Delta t}\right)-1\right)\mathrm{Lgp}\left(t,\mathrm{\Delta t}\right)+\mathrm{Bap}\left(t,\mathrm{\Delta t}\right)$$

Then, since as given above *Bp*(*t*, ∆*t*) approaches the positive value *Bap*(*t*, ∆*t*) whenever *Cap*(*t*, ∆*t*) approaches one, and since *Bp*(*t*, ∆*t*) varies continuously and does not changes signs, it must remain positive in all its variation range, then as a result *Lg*(*t*, ∆*t*) values will be systematically underestimated by their *Lgp*(*t*, ∆*t*) surrogates. Moreover, using inequality (26) we can find bounds for the variation of *Bp*(*t*, ∆*t*) that is, we have

(30) $$\mathrm{BLap}\left(t,\mathrm{\Delta t}\right)\leq \mathrm{Bp}\left(t,\mathrm{\Delta t}\right)\leq \mathrm{BLap}\left(t,\mathrm{\Delta t}\right)+\frac{p\left(t,\mathrm{\Delta t}\right)}{\mathrm{\Delta t}}\;\mathrm{Lgp}\left(t,\mathrm{\Delta t}\right)$$

where

(31) $$\mathrm{BLap}\left(t,\mathrm{\Delta t}\right)=\mathrm{Bap}\left(t,\mathrm{\Delta t}\right)-\mathrm{Lgp}\left(t,\mathrm{\Delta t}\right)$$

This result shows that the minimum possible value that *Bp*(*t*, ∆*t*) can attain is *BLap*(*t*, ∆*t*) and that whenever the inequality *p*(*t*, ∆*t*) ≥ ∆*t* holds the upper bound for its variation range will increase from the value *Bap*(*t*,∆*t*). Moreover this order relationship will induce wider variation ranges for *Bp*(*t*, ∆*t*) relative to the complementary case *p*(*t*, ∆*t*) < ∆*t*, therefore substantiating the claim by Short and Duarte [14] who stated that for an efficient application of the plastochrone method the length of the observation period ∆*t* must exceed the value *p*(*t*, ∆*t*) of the leaf plastochrone interval.

## Derivation of a formal relationship between leaf marking and plastochrone method assessments of eelgrass leaf growth rates

We now show that the addressed allometric framework can also be used to obtain a formal connection between leaf marking technique assessments and those obtained by means of the plastochrone method. With that aim let’s now denote by means of the symbol *Lgm*_*s*_(*t*, ∆*t*), the estimations of the *Lg*_*s*_(*t*, ∆*t*) rates of equation (3) which are obtained by means of the leaf marking technique. Lets also denote by means of *Lgm*(*t*, ∆*t*) the average of the *Lgm*_*s*_(*t*, ∆*t*) values over the number of shoots collected at time *t* + ∆*t* that is,

(32a) $$\mathrm{Lgm}\left(t,\Delta t\right)=\frac{\sum_{s}\;\mathrm{Lg}m_{s}\left(t,\Delta t\right)\;}{\;N\left(t+\Delta t\right)}.$$

And, if *Bm*(*t*, ∆*t*) denotes the related approximation bias we formally have

(32b) $$\mathrm{Lg}\left(t,\Delta t\right)=\mathrm{Lgm}\left(t,\Delta t\right)\;+\;\mathrm{Bm}\left(t,\Delta t\right).$$

Now, defining the ratio

(33) $$\lambda_{\mathrm{js}}\left(t,\mathrm{\Delta t}\right)=\frac{\Delta l_{\mathrm{js}}\left(t,\mathrm{\Delta t}\right)\;}{l_{\mathrm{js}}\left(t+\mathrm{\Delta t}\right)\;}$$

then, since ∆*l* is positive and bounded above by *l*_*js*_(*t* + ∆*t*),we have,

(34) $$0\leq \lambda_{\mathrm{js}}\left(t,\mathrm{\Delta t}\right)\leq 1$$

and, from equations (6) and (33) we get,

(35) $$\Delta \omega_{\mathrm{ajs}}\left(t,\mathrm{\Delta t}\right)\;=\alpha {\left(\Delta l_{\mathrm{js}}\left(t,\mathrm{\Delta t}\right)\right)}^{\beta }\left(\frac{\;1-{\left(1-\lambda_{l}\left(t,\mathrm{\Delta t}\right)\right)}^{\beta }}{\lambda_{l}{\left(t,\mathrm{\Delta t}\right)}^{\beta }}\right)$$

which rearranging leads to

(36) $$\Delta \omega_{\mathrm{ajs}}\left(t,\mathrm{\Delta t}\right)=\beta {\left(\Delta l_{\mathrm{js}}\left(t,\mathrm{\Delta t}\right)\right)}^{\alpha }+R_{\mathrm{js}}^{\mathrm{ma}}\left(\lambda ,t,\mathrm{\Delta t}\right)$$

where

(37) $$R_{\mathrm{js}}^{\mathrm{ma}}\left(\lambda ,t,\mathrm{\Delta t}\right)=\beta l_{\mathrm{js}}{\left(t+\mathrm{\Delta t}\right)}^{\alpha }\left(1-{\left(1-\lambda_{l}\left(t,\mathrm{\Delta t}\right)\right)}^{\beta }-\lambda_{\mathrm{js}}{\left(t,\mathrm{\Delta t}\right)}^{\beta }\right)$$

Hence, *Δω*_*ajs*_(*t*, *Δt*) can be split into two components: a term *β*(*Δl*_*js*_(*t*, *Δt*))^*α*^; the allometric expression of the biomass of the leaf length increment ∆*l*, plus a remainder, $R_{\mathrm{js}}^{\mathrm{ma}}\left(\lambda ,t,\mathrm{\Delta t}\right)$. The term *β*(*Δl*_*js*_(*t*, *Δt*))^*α*^, can be linked to the contribution of new leaf material to growth. And, since $\;R_{\mathrm{js}}^{\mathrm{ma}}\left(\lambda ,t,\mathrm{\Delta t}\right)$, depends on the whole leaf length span *l*_*js*_(*t* + *Δt*), this residual term can be taken as the contribution of mature leaf material to growth. Moreover, formally we must have

(38) $$\;\mathrm{Lg}m_{s}\left(t,\mathrm{\Delta t}\right)=\underset{j}{\sum }\frac{\beta {\left(\Delta l_{\mathrm{js}}\left(t,\mathrm{\Delta t}\right)\right)}^{\alpha }\;}{\mathrm{\Delta t}}$$

and assuming that equation (10) holds, equations (3) and (36) imply

(39) $$Lg_{s}\left(t,\mathrm{\Delta t}\right)=\mathrm{Lg}m_{s}\left(t,\mathrm{\Delta t}\right)+R_{s}^{\mathrm{ma}}\left(t,\mathrm{\Delta t}\right),$$

where

(40) $$R_{s}^{\mathrm{ma}}\left(t,\mathrm{\Delta t}\right)=\underset{j}{\sum }\frac{R_{\mathrm{js}}^{\mathrm{ma}}\left(\lambda ,t,\mathrm{\Delta t}\right)\;}{\mathrm{\Delta t}}$$

therefore, equations (15) and (39) yield,

(41) $$\mathrm{Lg}m_{s}\left(t,\mathrm{\Delta t}\right)=\;C_{s}^{\mathrm{ap}}\;\left(t,\mathrm{\Delta t}\right)\mathrm{Lg}p_{s}\left(t,\mathrm{\Delta t}\right)+R_{s}^{\mathrm{mp}}\left(t,\mathrm{\Delta t}\right)\;$$

where

(42) $$R_{s}^{\mathrm{mp}}\left(t,\mathrm{\Delta t}\right)=\underset{j}{\sum }\frac{\delta_{\mathrm{js}}\left(t,\mathrm{\Delta t}\right)\omega_{\mathrm{js}}\left(t+\mathrm{\Delta t}\right)\left(\left(I\left(j,3\right)-1\right)+\;\lambda_{\mathrm{js}}{\left(t,\mathrm{\Delta t}\right)}^{\beta }\right)}{\mathrm{\Delta t}}$$

then, averaging over the number of shoots collected at time *t* + ∆*t* we have that the *Lgm*(*t*, ∆*t*) and *Lgp*(*t*, ∆*t*) rates are related through,

(44) $$\mathrm{Lgm}\left(t,\mathrm{\Delta t}\right)=\mathrm{Cap}\left(t,\mathrm{\Delta t}\right)\mathrm{Lgp}\left(t,\mathrm{\Delta t}\right)+\mathrm{Bmp}\;\left(t,\mathrm{\Delta t}\right),$$

where

(45) $$\mathrm{Bmp}\;\left(t,\mathrm{\Delta t}\right)=\underset{s}{\sum }\frac{R_{s}^{\mathrm{mp}}\left(t,\mathrm{\Delta t}\right)\;}{N\left(t+\mathrm{\Delta t}\right)}$$

then as it was the case in equation (22), whenever the inequality,

(46) $$\mathrm{Bmp}\;\left(t,\mathrm{\Delta t}\right)>0$$

holds, the result of equation (44) implies that the *Lgm*(*t*, ∆*t*) assessments will be underestimated by the *Lgp*(*t*, ∆*t*) values.

## Corroboration of derived results using the present data

Using the present leaf data, we calculated the values for *in situ* leaf growth rates *Lg*(*t*, ∆*t*) by means of equation (4). The concomitant *Lgm*(*t*, ∆*t*) estimations produced by the leaf-marking technique were also obtained. Using the formula in Jacobs [4], we produced biweekly estimations of plastochrone-interval values. Plastochrone method proxies *Lgp*(*t*, ∆*t*) for the *in situ* leaf growth rates *Lg*(*t*, ∆*t*) were also calculated. The model of equation (5) was identified as consistent with the present data set, producing a coefficient of determination of 0.82. The values of the fitted parameters were *α* = 0.00002 and *β* = 1.3 similar to those previously reported for this site [34,35]. We then used these parameter values and associated leaf data to produce allometric estimations *Lga*(*t*, ∆*t*) for the *in situ* leaf-growth rates using equation (12a). An analysis of variance found significant differences between mean annual leaf-growth rates among methods (*F* = 17.95 *df* : 3, 112, *p* < 0.001) and an *a posteriori* Tukey test found non-significant differences among in situ values and those produced by the leaf marking method (*p* = 0.89), among in situ values and those obtained through the allometric method (*p* = 0.08), and among leaf marking values and those produced by using the allometric method *p* = 0.29. The Tukey test also showed that the mean annual leaf-growth rate obtained through the plastochrone method is statistically different from that linked to the *in situ* values, that calculated from leaf-marking data, and the one obtained from allometric projections (*p* < 0.001).

**Figure 1:**
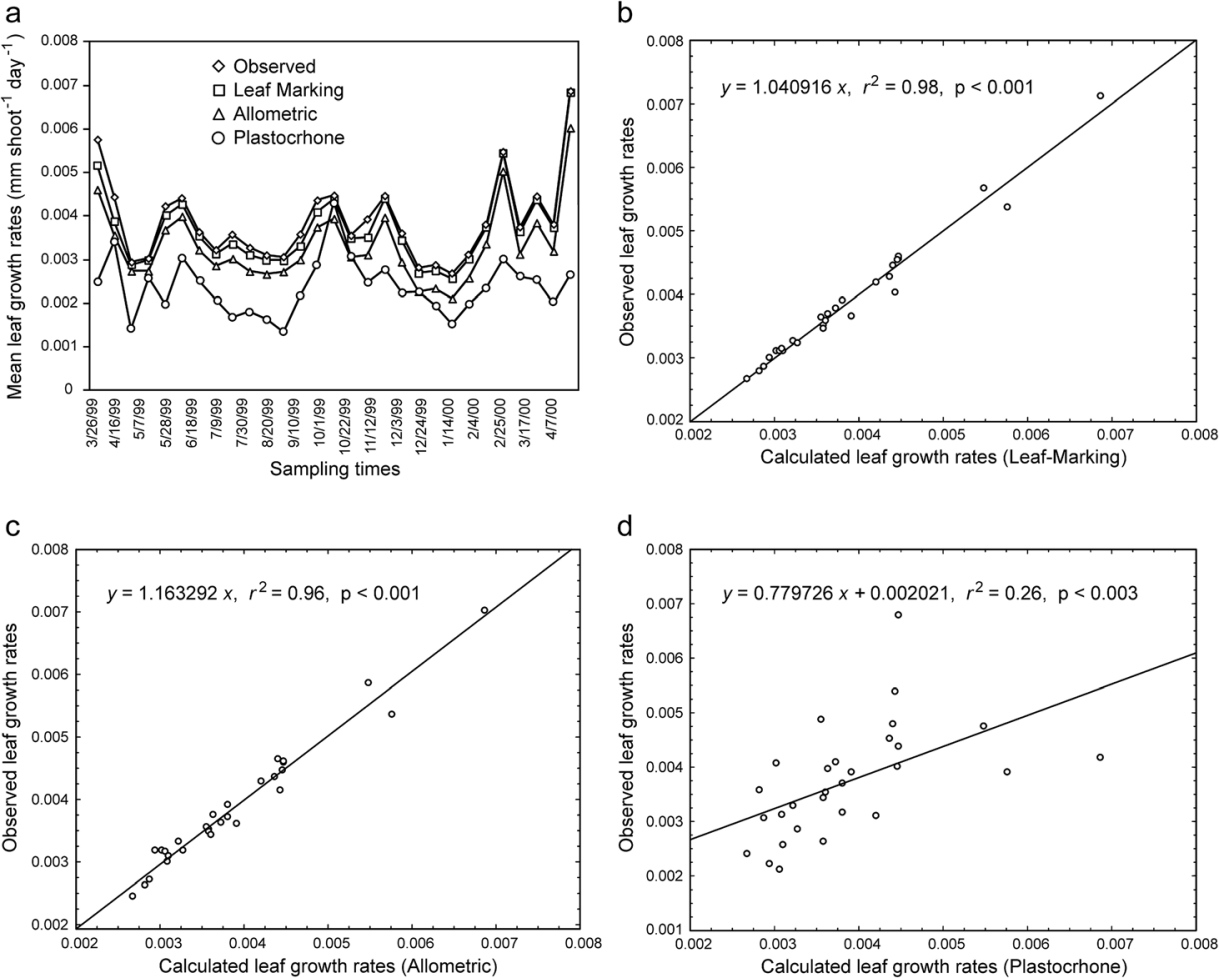
Comparison of observed and calculated leaf growth rates.

Figure 1a provides a direct comparison of measured *Lg*(*t*, ∆*t*) leaf growth rates and those calculated, by using the *Lgm*(*t*, ∆*t*), *Lga*(*t*, ∆*t*) or *Lgp*(*t*, ∆*t*) methods. It is shown that all these proxies produced positive bias in approximating the values for *in situ Lg*(*t*, ∆*t*) rates, but *Bm*(*t*, ∆*t*) was smaller relative to *Ba*(*t*, ∆*t*) and at the same time this bias was found to be smaller than *Bp*(*t*, ∆*t*). Moreover, since the bias term *Bmp*(*t*, ∆*t*) was also found to be positive, then equation (44) implies that the values of the plastochrone-method proxies *Lgp*(*t*, ∆*t*) underestimate the *Lgm*(*t*, ∆*t*) values obtained by means of the leaf-marking technique. Differences on the root mean squared error (RMSE) values on Table 1 suggest that while the leaf marking and allometric proxies could be expected to produce consistent projections of observed eelgrass leaf growth rates, the plastochrone method instead shows a relatively smaller reproducibility of the named rates.

**Table 1:** **Root mean squared error (RMSE) and concordance correlation coefficient (**$\hat{p}$**) values for comparison of observed leaf growth rates and corresponding proxies calculated by the leaf- marking, allometric or plastochrone methods**

**Criteria**	**Leaf marking**	**Allometric**	**Plastochrone**
RMSE	0.00021283	0.000586175	0.001700075
$\hat{\rho }$	0.9895	0.9831	0.5286
95% Confidence interval	(0.9776, 0.9951)	(0.9638, 0.9921)	(0.2010, 0.7498)

Moreover, an analysis of *Lgm*(*t*, ∆*t*) versus *Lg*(*t*, ∆*t*) values found a statistically significant regression through the origin; *Lg*(*t*, *Δt*) = 1.040 *Lgm*(*t*, *Δt*), *r*^2^ = 0.98, *p* < 0.001 (Figure 1b), also when we analyzed *Lga*(*t*, ∆*t*) versus *Lg*(*t*, ∆*t*) the same regression model was found highly consistent; *Lg*(*t*, *Δt*) = 1.163 *Lga*(*t*, *Δt*),  *r*^2^ = 0.96,  *p* < 0.001 (Figure 1c). Meanwhile, when we compared *Lgp*(*t*, ∆*t*) versus *Lg*(*t*, ∆*t*) no regression through the origin could be fitted. Instead only a poor fit; *Lg*(*t*, *Δt*) = 0.779 *Lgp*(*t*, *Δt*) + 0.002,  *r*^2^ = 0.26,  *p* < 0.003 could be produced (Figure 1d). Additionally, values of the Lin [36] Concordance Correlation Coefficient ($\hat{\rho })$ for reproducibility of the *Lg*(*t*, ∆*t*) rates by the methods addressed here (Table 1), reveal that both the leaf marking ($\hat{\rho }=0.9895)$ and allometric ($\hat{\rho }=0.9831)$ methods performed better than the plastochrone method ($\hat{\rho }=0.5286)$. Indeed we can observe that the bias *Ba*(*t*, ∆*t*), produced by the allometric method of equation (12a) is relatively smaller than that linked to plastocrone method assessments. Since the allometric approach is a simpler technique than leaf marking procedures [34,35], allometric projection is recommended as an effective method to reduce the bias associated to plastochrone method assessments.

## Discussion

Allometric scaling models of the form *Y* = *αX*^*β*^, where *Y* is a response and *X* is an explanatory variable, are commonly used in biological research. In this formulation, *α* is positive and is known as the normalization constant, while *β* is called the scaling exponent. From an empirical standpoint these allometic models have been fitted to many pairs of biologically traits *X* and *Y* in a highly consistent way, providing reliable methods for estimating variables that are difficult to quantify by using other variables that can be easily and directly measured. Empirical scaling models can be viewed as static relationships in which both the allometric exponent and the normalization constant take values that pertain to a particular data set. But beyond a purely empirical advantage, allometric approaches have demonstrated that prediction is not an unachievable aim in ecology. Indeed, allometric methods can be successfully used to predict roles and characteristics of organisms [37,38]. The predictive realm of allometric laws, moreover, is not circumscribed only to individual properties of organisms but can also apply to energy flows in whole ecosystems [39]. For example, the consistency of allometric relationships between body size and metabolism, which span 27 orders of magnitude in body mass for living organisms in both aquatic and terrestrial ecosystems, has provided a basis for the development of the metabolic theory of ecology [40].

The benefits of allometric methodologies in seagrass research were stressed by Duarte [41], who exemplified the use of static allometric models to grasp the implications of differences in plant size for productivity. For eelgrass in particular, Hamburg and Homann [28] and Echavarria-Heras *et al.*[35] used empirical allometric methods for the assessment of aboveground biomass. But allometric methodologies in eelgrass research are not limited only to empirical approaches for nondestructive appraisals of leaf biomass, but have also been used to formally substantiate empirical paradigms employed in eelgrass research [42]. They demonstrated that allometric scaling can provide criteria for evaluating the suitability of the leaf-biomass-to length ratio as a paradigm for nondestructive leaf biomass evaluations. And from a theoretical standpoint, it has also been established that allometric models can be linked to a paradigm for the indirect assessment of leaf growth rates in terms of simple measurements of leaf length or area [34].

It has been considered that conventional leaf-marking techniques produce biased estimations of eelgrass leaf-growth rates [11]. One fundamental objection states that, since these methods measure leaf growth by accounting only for the biomass of all the pieces of leaf material that have developed during the period between the marking and recovery of shoots, any leaf material produced below the reference point is not captured, and thus that portion of leaf production is missed. But even though the marking point could be placed on the sheath itself, as a way to avoid this inconvenience, the modified method still relies on newly produced leaf tissue, which is considered to be immature and flaccid. These tissues have a lower weight-to-length ratio than mature leaves [11], resulting in an underestimation of leaf growth.

The plastochrone method for eelgrass leaf growth rate estimations [4,11] was proposed in order to avoid the lack of representativeness of the leaf marking techniques. Unlike the leaf-marking techniques, the plastochrone method has been considered to produce simple, direct, nondestructive and unbiased estimations of eelgrass growth. Nonetheless, the reliability of this method has been largely vindicated at an empirical level, and practitioners have justified its application by asserting that this paradigm fully captures the growth pattern of eelgrass, in which new plant parts are incrementally produced while young tissue is simultaneously maturing.

The implementation of the plastochrone method is centered on the idea that the biomass of a single leaf representing mature tissue on a shoot, divided by the leaf plastochrone interval, provides a reliable substitute for the contribution of the shoot to the mean growth rate of leaves. But so far, a mechanistic explanation is still missing as to how a single leaf can capture the contribution to growth produced by all the leaves in a shoot over a given observation period. This is where allometric approaches can contribute to the formal substantiation of the method. Indeed, equation (15) was obtained by starting from the basic balance equation (3), then representing the contributions to growth of all the individual leaves on a shoot using the allometric representation of equation (6); and finally, by averaging over the number of retrieved shoots, we obtained equation (22). This equation relates the observed values of eelgrass leaf-growth rate *Lg*(*t*, ∆*t*) in a direct dynamical way to their proxy plastochrone method values *Lgp*(*t*, ∆*t*), thereby providing a formal authentication for this paradigm.

The balance equation (3) shows, moreover, that any fixed value of *Lg*_*s*_(*t*, ∆*t*) links all individual contributions to leaf growth in an essential way, with the biomass gained by a particular leaf being lost by the remaining ones. Hence, a single leaf would capture all the leaf biomass gained by a shoot over a given growing period, if and only if the biomass of the residual leaves in the shoot remained static, which is unsupported by the observed growth mode of eelgrass. This is indeed what inequality (19) properly expresses, and its validity formally explains that we can expect a downhill bias when plastochrone method calculations are used as proxies for observed leaf growth rates. This was in fact corroborated with the present data; the values obtained for the derived formulae confirmed that the plastochrone-method proxies underestimated the leaf-growth rates observed.

Likewise, the gains obtained from the use of allometric approaches are also shown by equation (37), which provides a device for the estimation of the contribution of leaf maturation processes to growth. But using the present theoretical settings, we can also address the controversy between the leaf marking and plastochrone methods for leaf-growth assessments. Indeed, equation (44) relates leaf-growth rate estimations obtained by means of the leaf marking technique to those obtained through the plastochrone method. And, the derived formulae set conditions under which it could be expected that plastochrone method approximations of eelgrass leaf-growth rates would systematically underestimate matching values obtained by using leaf-marking procedures. Indeed the condition set by inequality (46) was verified for the present data; then as a consequence, the plastochrone method proxies underestimated similar values obtained by means of leaf-marking methods. That is the inequality *Bp*(*t*, *Δt*) > *Bm*(*t*, *Δt*) holds through time, as shown in Figure 1. Further, using the present allometric framework, we formalized the claim of Short and Duarte [11] who stated that, for an efficient application of the plastochrone method, the length of the observation period ∆t must exceed the leaf plastochrone interval *p*(*t*, ∆*t*). And, although this condition was satisfied for the present data, we obtained  *Ba*(*t*, *Δt*) < *Bp*(*t*, *Δt*)  when approximating observed values. That is, the bias produced by the allometric method of equation (12a) was found to be smaller than that linked to plastocrone-method estimates. Furthermore, for this data the order relationship *Bm*(*t*, *Δt*) < *Ba*(*t*, *Δt*) < *Bp*(*t*, *Δt*) was maintained through time. It can be also observed from Figure 1 and from the high value of the associated Lin [36] Concordance Correlation Coefficient ($\hat{\rho })$ for reproducibility of the *Lg*(*t*, ∆*t*) rates through *Lgm*(*t*, ∆*t*) values (Table 1), that the contributions of leaves within the sheath, and of leaf maturation processes to growth, were not significant enough to reduce the effectiveness of the leaf marking technique.

And although a larger bias is associated with allometric projections than with leaf-marking techniques, the allometric approach is recommended over the plastochrone method because it is simpler and more precise (Table 1). Moreover our results support the conjecture that, taking into account the contribution to growth of all the leaves on an eelgrass shoot, as is done in both the leaf-marking technique and the allometric method provides a better proxy than the weight of a single leaf, as traditionally done in plastochrone method assessments. To this, we add that the lack of representativeness of this single-leaf-weight surrogate could explain the deviations shown in Figure 1.

Most methods used to assess leaf productivity in eelgrass have been vindicated at an empirical level. In particular, the plastochrone method has been favored over leaf-marking techniques because it is supposed to eliminate the alleged downhill bias linked to these assessments. This study confirms that a formal interpretation of assessment procedures is a crucial step in appraising their reliability, and we have shown how allometric methods can pave the way to achieve this important step. Formally, the plastochrone method will unavoidably produce biased estimations of the actual eelgrass leaf growth rate values. Empirical corroboration validated this inference, endorsing the view that allometric scaling relationships are not circumscribed only to empirical descriptions of the linkage between variables. Rather, they can be used to produce theoretical tools aimed towards the clarification of relevant issues in eelgrass research.

## Competing interests

The authors declare that they have no competing interests.

## Authors’ contributions

H conceived, designed and performed the research including the statistical analysis and interpretation of data. H has participated in manuscript drafting. E collected the data and performed the statistical tests. C performed the required mathematical proofs. O supervised the whole research and revised the manuscript critically both at statistical and formal levels. All authors read and approved the final manuscript.
